# Feasibility of Employing Semi-Hard Magnetic Materials for Hysteresis Magnetic Clutches in Railway Systems

**DOI:** 10.3390/ma18215044

**Published:** 2025-11-05

**Authors:** Paweł Pistelok, Marcin Adamiak

**Affiliations:** Faculty of Mechanical Engineering, Silesian University of Technology, Akademicka 2A, 44-100 Gliwice, Poland

**Keywords:** AlNiCoFe alloys, ferromagnetic materials, railway point machine, magnetic clutch, torque limiter, hysteresis clutch, microstructure, hysteresis loop, throwing force

## Abstract

This paper introduces innovative approaches to the design of railway point machines, with particular emphasis on the implementation of multi-component AlNiCoFe alloys, classified as semi-hard magnetic materials. A comprehensive review of existing mechanisms for mechanical force transmission—from the electric motor to the throwing bar—was conducted. The inherent limitations of conventional dry friction clutches, commonly used in current point machine designs, are critically analyzed. Furthermore, the feasibility of employing multi-component AlNiCoFe alloys as functional materials in hysteresis magnetic clutches is examined, with a view toward enhancing the reliability and performance of railway point actuation systems. A review of diagnostic methods for railway point machines was conducted to evaluate the potential application of a novel magnetic torque limiter as a means to eliminate maintenance activities typically required for systems utilizing dry friction clutches. Experimental research was performed on AlNiCoFe alloys employed as the hysteresis layer in the proposed torque limiter. Microstructural and compositional analyses were carried out using scanning electron microscopy (SEM), Energy Dispersive Spectroscopy (EDS), and X-ray Diffraction (XRD) to determine the crystallographic structure, chemical composition, and selected physical properties of the tested materials. The hysteresis loops of the tested materials were measured using a Vibrating Sample Magnetometer (VSM) over a wide temperature range. A prototype magnetic clutch, functioning as a torque limiter in a railway point machine, was developed and presented. The operational characteristics—specifically the throwing force as a function of time—were recorded for a railway point machine equipped with an electromechanical module incorporating the new magnetic torque limiter. The advantages of the proposed solution in terms of force transmission and overall system performance in railway point machine design were analyzed and discussed.

## 1. Introduction

Magnetic materials, particularly those classified as ferromagnetic, find wide application in industry [1,2,3,4,5]. One notable area of use is in magnetic devices that operate without active electromagnetic circuits—for example, magnetic couplings, which are generally categorized into hysteresis and eddy-current types [6]. The applications of such couplings are diverse, depending on the specific device in which they are employed. In this context, new intelligent magnetic materials can also be utilized. One example is magnetorheological fluid (MRF), which can transform from a liquid to a solid or semi-solid state within milliseconds when subjected to a magnetic field. MRF has been widely used in areas such as automotive systems, construction, vibration control, and ultra-precision machining [7,8,9]. A unique phenomenon occurring in magnetic clutches and brakes is the generation of eddy currents within conductive ferromagnetic materials. These currents are produced according to Faraday’s law of induction, with their magnitudes and directions governed by Ohm’s law and Lenz’s law, respectively. The generated eddy currents are eventually dissipated as heat energy. Consequently, the kinetic energy of the moving object is converted into electrical and thermal energy, which is removed from the system, resulting in deceleration [10].

The braking torque produced depends on the rotational speed. In the context of electromagnetic clutches, this phenomenon is not only a source of heat but also serves as the working principle of eddy-current brakes [10,11,12]. The induced eddy currents in the conductive layer generate their own magnetic field, which interacts with the primary magnetic field inside the device. This interaction produces a braking torque between the rotating and stationary parts of the brake [13,14].

Another type of magnetic clutch is the hysteresis clutch, whose braking torque ideally does not depend on rotational speed. However, in practice, achieving such independence is quite difficult [15,16,17]. One application area for magnetic couplings with hysteresis characteristics is in point machines—mechanical drives that safely switch railway turnouts [18]. These devices have a straightforward task: to move the blades of a turnout from one end position to the other. In essence, point machines act as actuators for railway switching and crossing systems, guiding trains from one track to another. This switching action must be completed within a few seconds (typically 3–8 s) without disturbances, halts, or jams. Predictive diagnostics is increasingly important for railway operators worldwide, primarily due to economic considerations. Predicting potential failures in turnouts or point machines enables planned maintenance, avoiding costly and unexpected disruptions in train traffic.

One approach to enabling fault diagnosis in railway point machines is the use of a digital twin—a digital representation of interdependent, critical components and systems. Although the concept of the digital twin originated in the military sector, it has since been widely adopted by leading industrial companies [19].

An alternative approach involves using vibration signal-based diagnostic methods, which offer advantages such as ease of data collection and strong resistance to interference. In this method, vibration signals are pre-processed using Variational Mode Decomposition (VMD) to achieve signal stationarity [20]. Following this, a multi-domain feature extraction technique is applied, which has been shown to be more effective than traditional single-domain methods. For the final diagnostic assessment and analysis, a Support Vector Machine (SVM) is employed. This method has demonstrated a diagnostic accuracy of up to 100%, with its superiority confirmed through multiple experimental comparisons [20,21,22,23,24,25,26,27,28].

Another diagnostic technique involves the use of Dynamic Time Warping (DTW) to address variations in the duration of RPM (railway point machine) movements, without requiring a training phase. Experimental results from RPM systems operated in Korea suggest that this training-free method can detect abnormal electric current patterns more accurately than conventional training-based approaches [29,30].

A further approach is the use of economic analysis for diagnosis, which focuses on identifying the most cost-effective maintenance policy for specific failure modes or system components [31].

**Figure 1:**
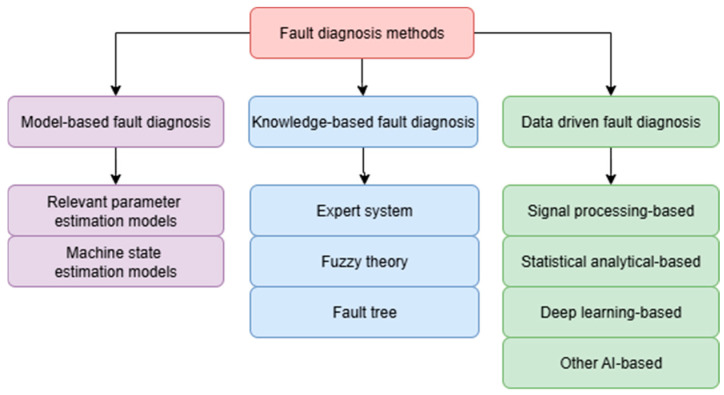
The categories of fault diagnosis methods [32].

The categories of fault diagnostic methods, as described in detail in [32], can be divided into three main types (Figure 1): model-based, knowledge-based, and data-driven fault diagnosis.

The diagnosis of point machines is increasingly being enhanced through the application of machine learning techniques [18]. Recently, several studies have reported promising fault diagnosis results specifically for railway applications [33,34,35], highlighting the critical role of maintenance in ensuring safe train operation. The railway point machine (RPM)—which consists of a motor, reduction gear, multiple bearings, drive-detection rods, and switches—is a key component responsible for changing the train’s travel direction. A crucial feature of RPMs is the inclusion of mechanical dry friction clutches, which serve to limit the torque transferred to the throwing bar. The typical design of a dry friction clutch used in railway point machines functions as a torque limiter. This design, commonly adopted in RPMs, is illustrated in the figure below.

**Figure 2:**
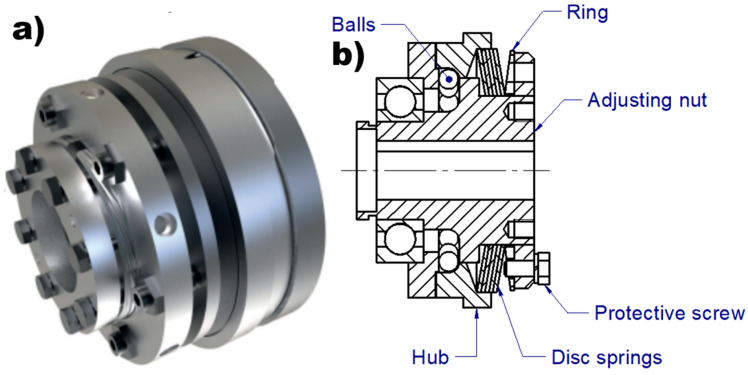
Dry friction clutch. (**a**) Type EAS-compact manufactured by MAYR company [39]. (**b**) cross section of EAS type clutch [39].

The working principle of a friction clutch is based on the axial pressure exerted by a spring, which generates a frictional force in the circumferential direction when relative motion between the driving and driven members begins to occur. If the torque resulting from this frictional force exceeds the required transmission torque, no slipping occurs, and power is effectively transmitted from the driving shaft to the driven shaft [36,37,38]. Mechanical clutches require regular maintenance and are subject to wear over years of operation. In dry friction clutches used in railway point machines—designed for long service lives of up to 25 years—the primary wear component is the ring and disc spring (Figure 2b). These wear-related issues are considered major disadvantages of dry friction clutches. An example of a mechanical solution used in point machines is the electrotechnical sliding unit, which incorporates a ball screw and a dry friction clutch to generate the throwing force required to switch the railway turnout. This system is illustrated in the figure below.

**Figure 3:**
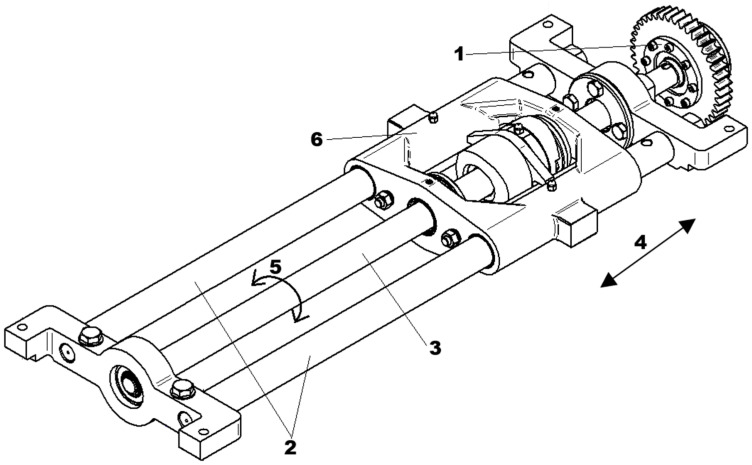
An example of a sliding unit with a dry friction clutch intended for a railway point machine, where 1—dry friction clutch with sprocket, 2—sliders, 3—ball screw, 4—direction of linear movement, 5—direction of ball screw rotation, and 6—saddle.

The sliding unit shown in the figure above provides linear movement of the saddle (6), which is mechanically connected to the throwing bar of the point machine (not visible in Figure 3). Mechanical torque is transmitted from the dry friction clutch via a sprocket (1) to the ball screw (3). The ball screw (3) is capable of rotating in both directions (5), enabling the saddle (6) to move linearly in both directions (4) along the sliders (2). This mechanical system, commonly used in electromechanical railway point machines, can be adapted to incorporate a magnetic torque limiter. The magnetic clutch offers the same functional parameters as the traditional sliding unit while addressing the drawbacks associated with dry friction clutches, such as wear and maintenance. A prototype of a hysteresis magnetic coupling has been implemented in a railway point machine, demonstrating stable operational performance during railway turnout switching under equivalent working conditions and requirements.

**Figure 4:**
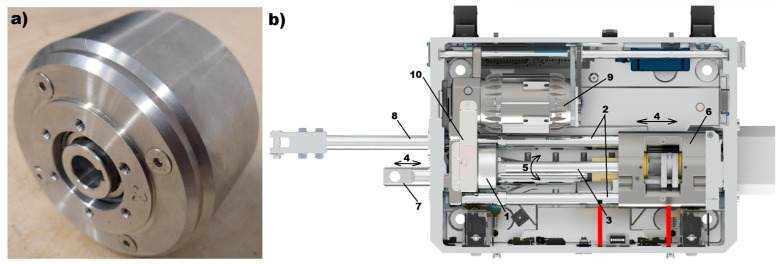
The magnetic clutch: (**a**) prototype prepared for tests and (**b**) prototype mounted inside of point machine, where 1—magnetic clutch, 2—sliders, 3—ball screw, 4—direction of linear movement, 5—direction of ball screw rotation, 6—saddle, 7—throwing bar of the point machine, 8—two detection bars (one under the other), 9—electric motor, and 10—gear train (sprockets).

The railway point machine equipped with a magnetic clutch (Figure 4b) generates the required throwing force using an electric motor (9), which transmits torque through a gear unit (10) and the magnetic clutch (1) to the ball screw (3). The rotation of the ball screw (5) produces linear movement of the saddle (6), which can move in both directions (4) along the sliders (2). The throwing bar (7) is mechanically connected to the saddle (6) and, on the opposite side, to the turnout blades (13). This mechanical linkage (14) enables the switching of the turnout. Additionally, the detection bars (8) and (16), which are moved by the turnout blades (13), provide feedback for monitoring the position of the blades.

**Figure 5:**
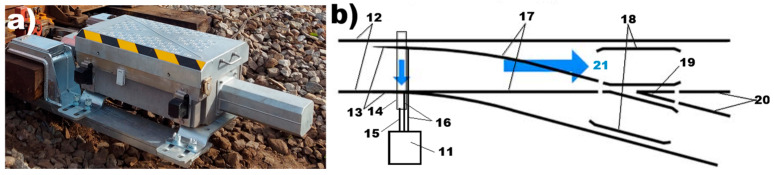
The railway point machine: (**a**) mounted in a railway turnout and (**b**) diagram of the railway turnout [40], where 11—point machine, 12—stock rails, 13—turnout blades, 14—throw rod, 15—throwing bar/rod, 16—detection bars/rods 17—closure rails, 18—guard rails, 19—frog, 20—frog rails, and 21—direction of the passing train.

The figure below presents a common turnout rail diagram with the direction configuration of a passing train “to the left”, as indicated by the blue arrow. The point machine, mounted on the turnout (Figure 5a) and indicated as position (11) in the diagram, is mechanically coupled (14) to the turnout blades (13).

## 2. Operating Characteristics of Magnetic Clutch Intended for Railway Point Machine

Taking into account the working conditions of the railway turnout (Figure 5b) and point machine, the presented magnetic clutch solution operates in two distinct modes: synchronous and asynchronous. In synchronous operation, mechanical torque generated by the electric motor (9) is directly transferred to the receiving unit—the ball screw (3). The rotation of the ball screw (5) produces the linear movement of the throwing bar (8) (15), enabling the switching action of the turnout.

The synchronous operation of the clutch ensures that the railway turnout switches within a specified time frame, which corresponds to the normal operating mode of the switch drive. The required switching time depends on the technical specifications of the turnout, the control system, and occasionally, local railway regulations. Typically, this time ranges from 3 to 8 s. For the clutch presented in this article, the target synchronous switching time is no more than 6 s under a mechanical load on the throwing bar of up to 4 kN.

The asynchronous operation of the clutch serves to limit the mechanical torque transmitted to the receiving system (ball screw) when the throwing bar (8) of the point machine becomes locked. Such locking can occur, for example, if the switch blade (13) is obstructed by an obstacle between the blade and the stock rail (12) during turnout switching. In this situation, the point machine must generate the required throwing force—up to a maximum of 5 kN for the magnetic clutch analyzed here—without causing damage. To meet these operational conditions, the magnetic torque limiter is designed to operate in a dual mode, accommodating both synchronous and asynchronous functions.

**Figure 6:**
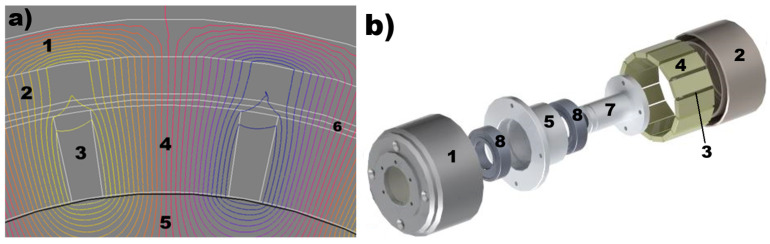
(**a**) Results of finite element calculations showing a 2D representation of magnetic flux lines within a segment of the torque limiter’s magnetic circuit. (**b**) Components of the magnetic clutch prototype: 1—outer yoke made from stainless steel, 2—hysteresis layer (material analyzed in this study), 3—air gap between permanent magnets, 4—neodymium permanent magnets (NdFeB), 5—inner yoke made from steel, 6—magnetic clutch air gap, divided into two layers, 7—shaft, 8—and bearings.

As part of the research to characterize the mechanical torque of a prototype magnetic clutch, a computational model was developed using the Altair Flux 2D/3D (v.2019.1) finite element analysis software. The results of these simulations, partially illustrated in Figure 6, indicate that the magnetic circuit was optimally designed to achieve the required magnetic induction levels in the clutch gap, as well as appropriate magnetic flux distribution throughout the various components of the magnetic circuit.

Figure 6 illustrates the magnetic flux lines in a section of the magnetic circuit of the prototype clutch shown in Figure 4, highlighting the correct orientation of the magnetic field (magnetic poles) within the magnetically semi-hard material. To verify the suitability of hysteresis materials for the required operational parameters of the railway point machine, extensive testing was conducted. This included microstructural analysis using scanning electron microscopy (SEM), compositional analysis with Energy Dispersive Spectroscopy (EDS), and X-ray Diffraction (XRD) to characterize the crystalline structure and material composition, as well as magnetic property evaluation using a Vibrating Sample Magnetometer (VSM).

## 3. Characteristics of Magnetic AlNiCoFe-Based Alloys

AlNiCo alloys represent a key class of permanent magnetic materials distinguished by their unique thermal and structural properties. Their development dates back to 1931, when T. Mishima and his team discovered that an alloy composed of iron, nickel, and aluminum exhibited coercivity significantly higher than that of the best steel magnets available at the time [41]. These alloys are widely used across various industries, including computing, office consumer products, automotive and transportation, electronic devices, manufacturing equipment, as well as medical and military applications. The magnetic behaviour of these materials is closely tied to the control of their phases and the use of nanoparticles, which enable the tailoring of either soft or hard magnetization states depending on the thermomechanical treatments applied during sintering processes that follow the initial phase formation [3,4,42]. One of the most notable characteristics of AlNiCo alloys is their exceptional temperature stability. Numerous studies confirm that these alloys can operate at temperatures up to 550 °C while maintaining stable magnetic properties [43,44]. Furthermore, the temperature coefficient of remanent induction (Br) for AlNiCo alloys is approximately −0.02%/°C, one of the lowest values among available magnetic materials [4].

**Figure 7:**
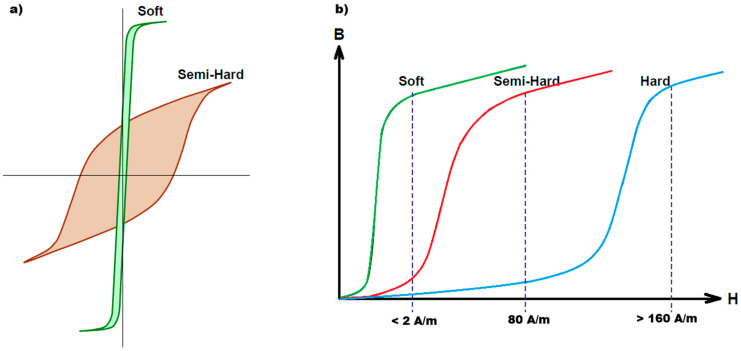
An example of hysteresis loops: (**a**) typical ferromagnetic materials, (**b**) The hysteresis loops of ferromagnetic materials divided into three groups.

Detailed studies on the impact of chemical composition on the thermal stability of AlNiCo alloys reveal that both cobalt and nickel contents play significant roles in determining these properties. Nickel, as a major component of the matrix phase, affects the morphology of the spinodal decomposition as well as the characteristics and proportions of the resulting α_1_ and α_2_ phases [43,44]. Increasing the nickel content generally leads to a higher proportion of the NiAl-rich phase within the alloy’s microstructure, which enhances the overall thermal stability. Additionally, an optimally chosen nickel ratio encourages the development of a favourable microstructure, where the α_1_ phase—responsible for the alloy’s magnetic properties—is uniformly dispersed throughout the matrix. This uniform dispersion ensures high remanence and coercivity [44,45,46]. The characteristic hysteresis loop of AlNiCo alloys features a nonlinear demagnetization curve that remains stable across a wide temperature range. Temperature hysteresis studies indicate that AlNiCo alloys maintain consistent hysteretic behaviour between −195 °C and 400 °C [47]. This thermal stability is especially important in magnetic clutch applications, where predictable and reliable magnetic properties are required regardless of ambient temperature conditions. In terms of temperature stability, the objective is to tailor the AlNiCo alloy to maintain its performance within the saturation zone while preserving key parameters such as coercivity (Hc) and remanent induction (Br) under varying temperature conditions. The absence of deviations in the magnetic saturation zone of the material across selected ambient temperature ranges is critical for ensuring both the product’s life cycle and the reliable operation of devices made from the alloy developed in this research. The graph below illustrates typical hysteresis loops (Figure 7a) for ferromagnetic materials, categorized into three groups (Figure 7b): soft, semi-hard, and hard magnetic materials.

The thermal stability of AlNiCo alloys stems from the spinodal microstructure formed during heat treatment through spinodal decomposition. According to the literature, the optimal spinodal structure consists of rod-like α_1_ phase particles, measuring 30–45 nm in diameter, embedded within an α_2_ phase matrix [45,48]. This mosaic-like microstructure is key to providing magnetic stability across a wide temperature range. The properties observed and analyzed in AlNiCo alloys highlight their strong potential for use in magnetic clutches designed for railway point machines, which operate in demanding and harsh environments.

## 4. Microstructures and Diffractograms

The exanimated material samples were selected in scope of possibility used in cylindric hysteresis clutch. Such design requires a ring shape of hysteresis layer. Alnico alloys, commonly used for magnetic application, are well-suited for casting due to their ability to be formed into complex shapes. Cast Alnico magnets offer high magnetic strength and good temperature stability, but they also have limitations like low coercivity (making them susceptible to demagnetization) [1,2,3,4,42].

High-resolution scanning electron microscopy (HRSEM) was performed using a HRSEM SUPRA 35 (Zeiss, Oberkochen, Germany). The system is equipped with a secondary electron detector (SE), a backscattered electron detector (QBSD), and an energy-dispersive X-ray spectrometer (EDS). Microscopic observations were performed at an accelerating voltage of 20 kV. Samples 1, 2, 3 were examined using a secondary electron detector (SE). The SEM examinations were supplemented with EDS analysis of the chemical composition.

Phase composition studies were performed on a Panalytical X’Pert PRO MPD X-ray diffractometer equipped with a copper/cobalt anode X-ray tube (λKα = 0.154 nm) and a PIXcel 3D detector (Malvern, UK). Diffraction patterns were recorded using Bragg–Brentano geometry in a 2Theta angle range of 20–110°, with a 0.05° step and a counting time of 100 s/step. Qualitative X-ray phase analysis was performed using HighScore Plus software (v. 3.0e) and the dedicated PAN-ICSD database of inorganic crystal structures.

### 4.1. Sample No 1

**Figure 8:**
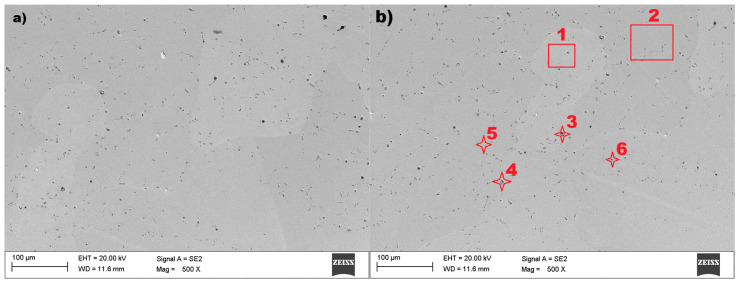
The microstructure of sample no 1: (**a**) SEM result and (**b**) measuring points 1–6 for the EDS method.

Based on the literature review, the tested materials were recognized as AlNiCo alloys and examined by using scanning electron microscopy (SEM) and Energy Dispersive X-ray Spectroscopy (EDS). Both methods were used to verify the microstructure and elemental composition of the examined material samples. The microstructure of sample no 1 is presented in Figure 8.

**Figure 9:**
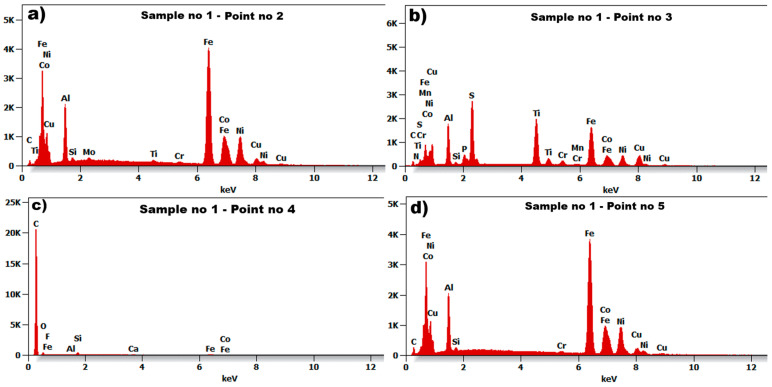
The Spectrum of Energy Dispersive Spectroscopy (EDS) for sample no 1: (**a**) point no 2, (**b**) point no 3, (**c**) point no 4, and (**d**) point no 5.

The microstructure of the tested material shows (Figure 8) the size and orientation of the grain. The grain diameter of sample no 1, presented in Figure 8a, is barely visible with a regular shape. The diameter of the visible grain can be set to within the range of 100–150 nm. Relatively small inclusions were found on the surface of the tested sample no 1, and the imaged material grains can be classified as heterogeneous. The EDS spectrum for selected points 2, 3, 4, and 5, marked in Figure 8b, are presented in Figure 9.

The six examined regions (Figure 8b) of sample no 1, point 3 had Ti, S, and C (point 4, Figure 8b) precipitates throughout the cast microstructure, as shown by the small dark grey faceted phases. The analysis of the EDS spectrum, presented in Figure 9a,d, identify and quantify the elemental composition of the tested material. The results of energy dispersive spectroscopy for sample no 1 indicate that the main components of the tested material—iron Fe, cobalt Co, nickel Ni, aluminum Al—are typical for AlNiCo alloys.

**Figure 10:**
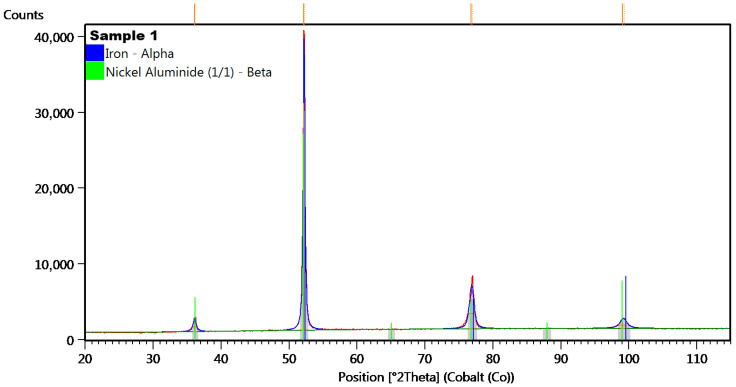
The XRD diffractogram for sample no 1.

To determine the arrangement of atoms within a crystal lattice, including lattice parameters and unit cell dimensions, the material was tested using the X-ray Diffraction method. This non-destructive analytical technique was used to characterize the structure and composition of crystalline materials. The diffraction pattern was analyzed to determine the crystal structure, phase identification, and other properties of the material (Figure 10).

**Figure 11:**
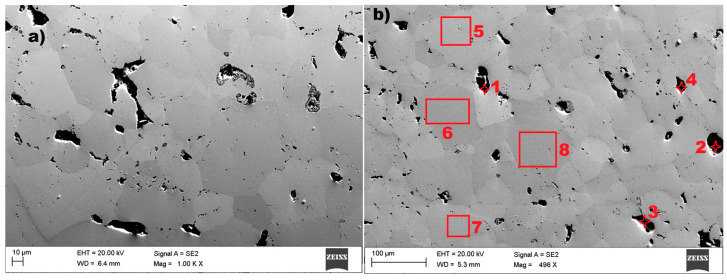
The microstructure of sample no 2: (**a**) SEM result and (**b**) the measuring points 1–8 for the EDS method.

In the diffractogram of sample no 1 (Figure 11), the diffraction lines were identified at angular positions and with an intensity distribution characteristic of the α-iron crystal phase (cubic lattice I m −3 m). The analyzed diffraction pattern also recorded diffraction lines from Ni_3_Al phases.

### 4.2. Sample No 2

In the case of sample no 2, also examined by using scanning electron microscopy (SEM) and Energy Dispersive X-ray Spectroscopy (EDS), the result are presented in the figures below. The microstructure of the tested sample are presented in Figure 11.

**Figure 12:**
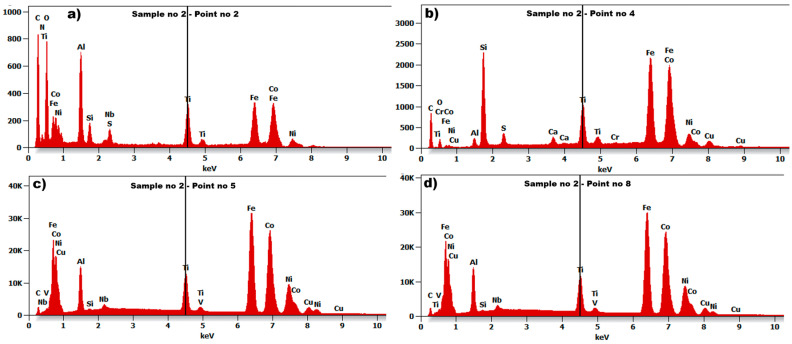
The Spectrum of Energy Dispersive Spectroscopy (EDS) for sample no 2: (**a**) point no 2, (**b**) point no 4, (**c**) point no 5, and (**d**) point no 8.

The microstructure of the tested material shows (Figure 11) the size and orientation of the grain. The image reveals grains with irregular shape and a large variation in grain size. The diameter of the visible grain can be set from 10 µm to even 150 nm. A relatively large number of inclusions were found in the tested sample no 2, which is proof of the cast’s low quality. The visible grain of the material can be classified as a structure typical of AlNiCo alloys. The EDS spectrum for the selected points 2, 4, 5, and 8, marked in Figure 11b, are presented in Figure 12.

The seven examined regions (Figure 11b) give comparable results, showing the results for four of them (Figure 12). Sample no 2, point 2 had C, Ti, S, and Si (point 4, Figure 12b) precipitates throughout the cast microstructure, as shown by the small dark grey faceted phases in Figure 12b. The analysis of the EDS spectrum, presented in Figure 12c,d, identify and quantify the elemental composition of the tested material. In the tested sample no 2, titanium (Ti) was discovered in measuring points 2–8 in Figure 11b, with comparable amount in relation to aluminum (Al). The results of energy dispersive spectroscopy for the examined sample indicate that the main components of the tested material designated as sample 2—iron Fe, cobalt Co, nickel Ni, and aluminum Al—are typical for AlNiCo alloys. The presence of copper (Cu) was also detected, as in sample no 1. To determine the crystal lattice, including lattice parameters and unit cell dimensions, the material was also tested by the X-ray Diffraction method. The diffractogram of sample no 2 is presented in the figure below.

**Figure 13:**
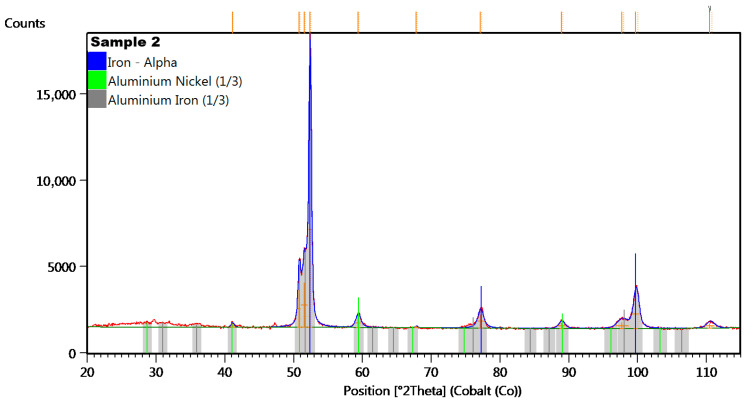
The XRD diffractogram for sample no 2.

In the diffractogram of sample no 2 (Figure 13), the diffraction lines were identified at angular positions and with an intensity distribution characteristic also of the α-iron crystal phase (cubic lattice I m −3 m). The analyzed diffraction pattern also recorded diffraction lines from Ni_3_Al phases and iron-aluminum Fe3Al.

### 4.3. Sample No 3

**Figure 14:**
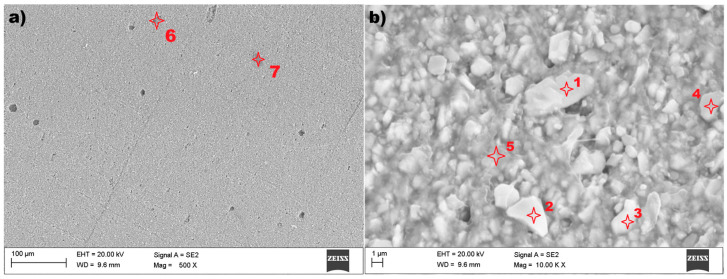
The microstructure of sample no 3: (**a**) SEM result and (**b**) measuring points 1–7 for EDS method.

In the case of sample no 3, also examined using scanning electron microscopy (SEM) and Energy Dispersive X-ray Spectroscopy (EDS), the results are presented in the figures below. The microstructure of the tested material is presented in Figure 14.

**Figure 15:**
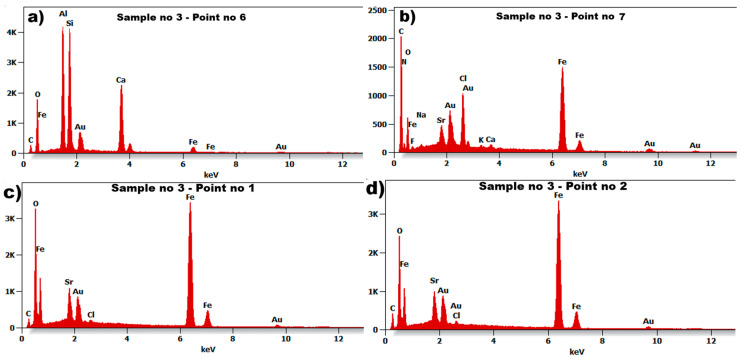
The Spectrum of Energy Dispersive Spectroscopy (EDS) for sample no 3: (**a**) point no 2, (**b**) point no 4, (**c**) point no 6, and (**d**) point no 7.

The test results of scanning electron microscopy for sample no. 3 showed an unexpected microstructure in relation to previous tested materials. The image (Figure 14a) reveals a structure without visible grains at a magnification of 500×. After magnifying the surface twenty times more (10,000×), the structure of sample 3 can be visible—Figure 14b. It is shown that the domain structure of the particle is approximately plate-like. The particles have an irregular shape, and their size can be identified ranging from 0.5 µm up to 5 µm. A relatively small number of inclusions were found in the tested sample no 3, which is presented in Figure 14a. The EDS spectrum for the selected points 1, 2, 6, and 7 (marked in Figure 14) are presented in Figure 15.

The seven examined points (Figure 14) give comparable results, showing the results of only four of them (Figure 15). Sample no 3, point 6 (Figure 15a) had Al, Si, Ca, and C (point 7, Figure 15a) precipitates throughout the cast microstructure. The analysis of the EDS spectrum identified iron Fe and oxygen O were at measuring points 2–7 in Figure 14. The results of energy dispersive spectroscopy for the examined sample indicate that the main components of the tested material designated as sample 3 are iron Fe, oxygen O, and gold Au, with strontium Sr in similar amounts. The lack of copper (Cu) was detected in comparison to the evaluated samples 1 and 2.

To determine the crystal lattice, including lattice parameters and unit cell dimensions, sample 3 was tested by X-ray Diffraction method. The diffractogram of sample no 3 is presented in the figure below.

**Figure 16:**
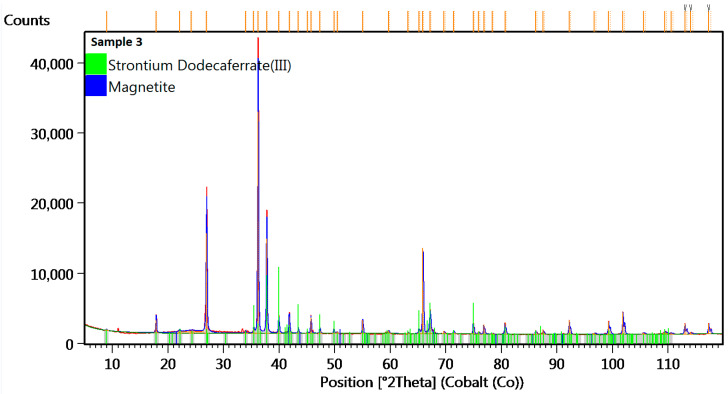
The XRD diffractogram for sample no 3.

The diffractogram of sample no 3 (Figure 16) shows diffraction lines identified at angular positions and with an intensity distribution characteristic of the strontium dodecairon (III) oxide (Fe12O19Sr1) crystal phase (hexagonal lattice P 63/m m c). The analyzed diffraction pattern also recorded diffraction lines from Magnetite (Fe3O4) crystal phase (cubic lattice F d −3 m). The analyzed diffraction pattern was recorded using a cobalt anode.

## 5. Hysteresis Loops

**Figure 17:**
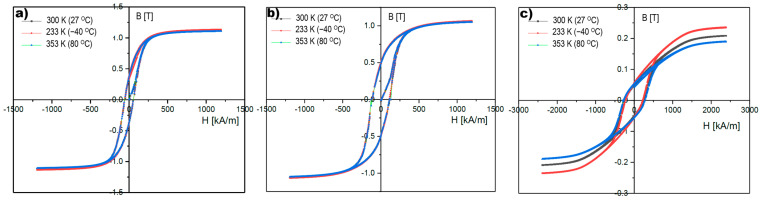
The hysteresis loop for (**a**) sample no 1, (**b**) sample no 2, and (**c**) sample no 3.

To develop an optimal magnetic clutch, seven AlNiCo alloys were tested to verify their magnetic properties, which are most important for providing the required operational parameter for the point machine, especially in wide temperature range from −40 °C to 80 °C. Based on the test results received by using the VSM method, the hysteresis loops in three temperature values were measured and are presented in Figure 17. The VSM method is based on Faraday’s law, according to which an electromotive force (EMF) is induced in a conductor by a time-varying magnetic flux. In the VSM method, a sample magnetized by a uniform magnetic field is vibrated sinusoidally at a small constant amplitude against stationary sensing coils [49].

**Figure 18:**
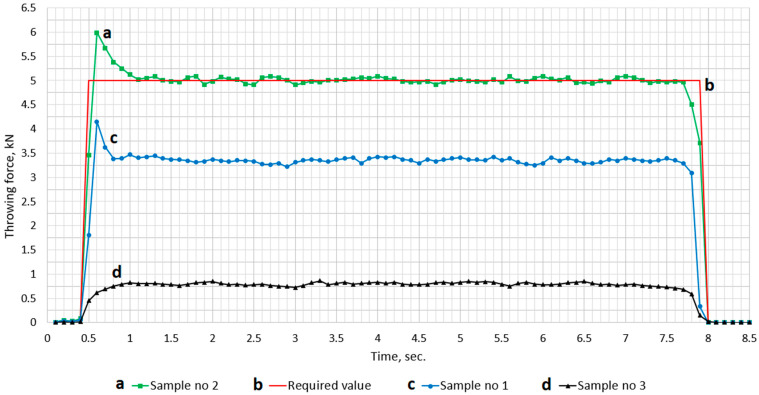
The throwing force of the railway point machine equipped in a prototype of magnetic clutch with three tested materials as the hysteresis layer: (**c**) hysteresis layer made from the material of sample no 1, (**a**) hysteresis layer made from the material of sample no 2, and (**d**) hysteresis layer made from the material of sample no 3, (**b**) required course of throwing force during point machine movement.

Based on the results received by the SEM+EDS+XRD methods, three material samples were selected to check their magnetic properties. These selected materials show high potential for use in railway point machine in terms of formability and scalability in different (other) magnetic circuits, which can be required in case of different railway point machines. Taking into account the identified microstructures of the tested materials, samples no 1 and 2 are AlNiCo alloy with optimal temperature stability of induction B and field strength saturation H, as shown in Figure 17a,b. The saturation values do not exceed B = 1.1 T and H = 1250 kA/m. These values determine the parameters of the magnetic circuits required to provide demagnetisation process inside the hysteresis layer. The important parameters that were measured are remanent induction Br and coercivity Hc. The noticed values for sample 1 were Hc ≈ 50 kA/m and Br ≈ 0.34 T, and for sample no 2, Hc ≈ 110 kA/m and Br ≈ 0.49 T. In the case of the third tested material, which belongs to a group of cheap composite materials with a polymer matrix containing magnetic compounds, the saturation values are not stable across the measured temperature values, as shown in Figure 18d. The coercivity and remanent induction were measured Hc ≈ 270 kA/m and Br ≈ 0.05 T.

## 6. Discussion—Results Validation

The railway area is a very broad branch of industry where innovative solutions could be implemented. One original approach in railway point machine (RPM) design is the implementation of a prototype magnetic clutch, which provides stable RPM operational parameters in a wide range of temperature (−40 °C – 80 °C) and harsh vibration (vertical—130 m/s^2^, transversal—50 m/s^2^, longitudinal—90 m/s^2^) and shock (300 m/s^2^ during 8.0 ms and 800 m/s^2^ during 2 ms) environments while maintaining a relatively lowest cost of production for such a device. Based on the solutions used in dry friction clutches for railway point machines, the new magnetic torque limiter will be maintenance-free and provide stable operation without adjustment during the lifetime of the railway point machine, which is 25 years. The magnetic clutches are also offered in the market, but their adaptation in the scope of railway industry requirements is expensive and economically unprofitable. The main goal of such a design is the lowest possible production cost of the magnetic clutch, which suggests the use of common materials such as AlNiCo alloys, permanent magnets (NdFeB, SmCo, SrFe), and steel. With such requirements, the prototype of the magnetic clutch was designed using finite element method calculations, with a perspective to using the materials discussed in this paper—samples no 1, 2, and 3—as the hysteresis layer. The calculation results of the optimal arrangements of flux lines in the magnetic circuit (Figure 6) was the first stage in which the prototype was built, as presented in Figure 4a. The prototype torque limiter was designed with taking into account the dimensional limits imposed by the railway point machine and the required operational parameters in terms of the transferred and limited forces on the throwing bar of the point machine (for details, see Section 2). The prototype, prepared for tests with three materials (sample 1, 2, and 3) as the hysteresis layer, was mounted inside the railway point machine (Figure 4b) and verified with respect to operational parameters.

Based on the test results of the analyzed microstructure for samples no 1 and 2, the grain diameter in both cases is very similar, but the grain of sample no 1 is barely visible in relation to sample no 2. The material of sample no 2 is characterized by relatively high porosity. In the case of sample no 1, the main chemical composition was (Figure 9) aluminum Al, nickel Ni, and cobalt Co, with a large amount of iron Fe. In sample no 2, the chemical composition was aluminum Al, nickel Ni, cobalt Co, and titanium Ti, also with large amount of iron Fe.

The microstructure of sample no 3 is incomparably a different structure compared to the materials of samples 1 and 2. The grain size is relatively small and is from about 0.5 um up to 5 µm. The domain structure of the particles is approximately plate-like. The main chemical composition of sample no 3 was strontium Sr, oxygen O, gold Au, and a large amount of iron Fe.

Inclusions were noticed in all three tested materials, and for sample no 1, these were elements of carbon C and sulphur S. In the case of sample no 2, inclusions of sulphur S, silicon Si, and carbon C elements were discovered. The microstructure of the material in sample no 3 reveals a relatively small amount of inclusions in comparison to samples 1 and 2, which contain elements of aluminum Al, silicon Si, calcium Ca, and carbon C.

Based on the analyzed positions and intensities of the peaks in the XRD diffractogram in Figure 10 (sample no 1) and Figure 13 (sample no 2), the researchers can determine that the samples contain α-Fe, which confirms the body-centred cubic (BCC) structure and provides stability at room temperature and atmospheric pressure. The present of the alpha iron phase indicates susceptibility to oxidation and corrosion in moist or acidic environments, as it reacts with oxygen, forming iron oxide (rust) when exposed to moisture [50,51]. The alpha iron is the predominant phase in low-carbon steels and is critical in steel-making processes, but it exhibits good fatigue properties, making it suitable for applications in harsh vibration environments.

Analysis of the diffractograms for samples 1 and 2 indicated the presence of nickel aluminide phases. This term generally denotes one of two prevalent intermetallic compounds, Ni_3_Al or NiAl, though it is sometimes used generically for any nickel-aluminum alloy. The authors of reference [42] noted that the inclusion of Ni facilitated the formation of more organized structures. These alloys are extensively utilized due to their advantageous properties, such as high strength retained at elevated temperatures (above 800 °C), low density, corrosion resistance, and simplicity of manufacturing [52]. The Ni_3_Al compound, in particular, provides these alloys with high strength and resistance to creep at temperatures up to 0.7–0.8 of its melting point [52,53]. NiAl also demonstrates excellent characteristics, possessing a lower density and higher melting temperature than Ni_3_Al, as well as good thermal conductivity and resistance to oxidation [53]. These qualities make it a compelling choice for specific high-temperature applications, including the railway industry. The disadvantage associated with both compounds is their considerable brittleness at room temperature. Ni_3_Al specifically remains brittle at high temperatures as well [52]. To resolve this issue, Ni_3_Al can be rendered ductile by manufacturing it in a single-crystal form rather than in its polycrystalline state [52].

The diffractogram for sample no 2 reveals aluminum iron (FeAl), which can be used as deoxidizers in steelmaking or as reducing agents [54,55]. The authors of paper [56] also concluded that the presence of Fe-Al in the material during production should be selected optimally. Exceeding the minimum amount of iron necessary for full surface coverage led to the formation of a thick shell, which obstructed galvanic corrosion occurring at the surface. In contrast, an insufficient amount of iron resulted in only partial coverage of the aluminum surface. The efficacy of the bimetallic Fe/Al outer surface declined in areas lacking iron coverage.

The discovered microstructure of the tested material in sample no 3 showed similarity to the barium ferrite microstructure [57,58]. The discovered (XRD) compound FeOSr (strontium dodecairon oxygen) is a hard magnetic material with high coercivity and is used in various industrial applications, including electric motors. The good magnetic properties and flexibility of this material indicate a high potential for use in other applications (magnetic circuits) intended for railway point machines.

Based on the conclusion of the authors of paper [57], the magnetic domains of barium ferrite particles less than 10 to 1 µm in size were identified (by SEM). This compound is used to increase the magnetic properties of alloys. On average, the domain count within a particle diminishes as the particle size is reduced. For particles below approximately 2 µm, they are typically composed of two domains. It has been shown that the domain configuration of such two-domain particles is plate-like. Furthermore, the particle morphology is identified as a double hexagonal pyramid, roughly aligning with the c-plane. A successful observation of a single-domain particle measuring 1.3 µm has also been reported.

The tests performed in the scope of publication [58] highlight a few aspects. The coercive force of the studied barium ferrite is dependent on their annealing temperature, and the best coercive force was obtained for powders annealed at temperature of 950 °C., which is important information in the context of using materials, whose composition is based on barium ferrite, in different railway application, e.g., magnetic torque limiter for other types of point machines.

The different saturation values depending on temperature require different flux values in the magnetic circuit, which disqualifies the third material (sample no 3) from further tests and its use in the prototype magnetic cutch intended for railway point machines. The instability of saturation parameters, too low values of remanent induction, and too high value of coercivity do not provide the required operational parameters for the point machine. In the case of samples no 1 and 2, the magnetic properties were noticed as acceptable, especially the stability especially at the limits of the required operating temperature range (Figure 17).

The graph in Figure 18 presents the course of the throwing force which is required (red one) to fulfil railway point machine requirements. The value of the throwing force was measured at the throwing bar (7) eye, shown in Figure 4b, by inserting the measuring pin into the throwing bar eye. The throwing bar (7) is blocked somewhere near the middle of the cycle movement; the turnout switching and blocking time depend on the control system configuration the particular turnout and is within the range from 7 to 10 s. The image reveals a very stable course of throwing force value for all three tested materials. At the beginning of the throwing force course, a peak value was observed: up to 4.2 kN for sample no 1 and 6 kN for sample no 2. This point also marks the start of the asynchronous duty point of the magnetic clutch, when the demagnetization process is starting in hysteresis layer. The value stabilizing after ~500 ms in both cases. Sample no 3 generates a very low value of a throwing force and does not fulfil the requirement of the railway point machine. The hysteresis loops (Figure 17), measured in the required temperature range (−40 °C, 80 °C), confirm the stability of saturation parameters (B, H) for samples no 1 and 2, but only sample no 2 is acceptable, because its measured throwing force (Figure 18) fulfils the railway point machine requirement. The most interesting observations are the courses for samples no 1 and 2, which show different values of the throwing force despite similar microstructure, chemical composition, and diffractograms. The discovered differences between samples no 1 and 2 show direction to optimization, which can be used in further research or other railway applications.

## 7. Summary and Conclusions

Based on the test results of the microstructures and diffractograms the tested materials, there is high potential for their use as a hysteresis layer in magnetic clutches intended for railway point machines. The physical properties like corrosion resistance, mechanical strength, and magnetic features are entering the area of application of AlNiCo alloys in the railway industry. The highest stability of magnetic parameters (saturation values of B and H) was noticed for samples no 1 and 2 (Figure 17a,b), which contain the elements Al, Ni, Co, and Ti and a large proportion of Fe, consistent with AlNiCo alloys. Despite good mechanical features and flexibility, the material in sample no 3 was unstable in low and high temperatures (−40 °C, 80 °C), in terms of magnetic properties (Figure 17c). These discrepancies in the saturation values of B and H disqualify it for use in a magnetic clutch intended for railway point machines.

Based on the conclusion from the analysis results of the mechanical torque characteristics (Figure 18) of the magnetic clutch with tested materials used as the hysteresis layer, the optimal material is sample no 2. This material provides the required operational parameters for the magnetic clutch. The operational parameters of the railway point machine, equipped with a magnetic clutch with AlNiCo alloys as the hysteresis layer, will stably work in the required temperature (−40 °C, 80 °C), with acceptable immunity for harsh mechanical shocks and vibration in the railway area.

In the case of sample no1, the throwing force of the point machine with the magnetic torque limiter was too low and not acceptable. The reason for such a result is the narrower hysteresis loop of sample no 1 in relation to the wider hysteresis loop of sample no 2, in which the operational parameters of the railway point machine were acceptable (Figure 18).

## Data Availability

The original contributions presented in this study are included in the article. Further inquiries can be directed to the corresponding authors.

## Abbreviations

The following abbreviations are used in this article:

EDS	Energy Dispersive Spectroscopy
SEM	Scanning Electron Microscopy
XRD	X-Ray Diffraction
RPM	Railway Point Machine
VSM	Vibrating Sample Magnetometer method.
EMF	Electromotive Force
AlNiCoFe	Alloy with elements Al, Ni, Co, Fe
